# Ovarian stimulation modalities in poor responders

**DOI:** 10.3906/sag-1905-179

**Published:** 2019-08-08

**Authors:** Zehra Sema ÖZKAN

**Affiliations:** 1 Department of Obstetrics and Gynecology, School of Medicine, Kırıkkale University, Kırıkkale Turkey

**Keywords:** Poor ovarian response, cycle cancellation, DHEA, double stimulation

## Abstract

In a group of IVF/ICSI cycles, despite the appropriate ovarian stimulation, the number of oocytes collected is below the expected value. This condition is defined as poor ovarian response (POR) to stimulation. POR brings the risk of cycle cancellation with an estimated rate of 20%. Infertility experts are trying to improve cycle outcomes of POR cases with multiple modifications. This review article will present the latest modifications on the management of POR. The studies performed for improving cycle outcome in POR cases were evaluated and their notable results were presented. The first intervention among infertility specialists is to make a standard definition for POR. The BOLOGNA criteria and the subsequent POSEIDON group definitions are the latest updates in POR management. GnRH antagonists, estradiol priming, double stimulation, letrozole administration, DHEA, and herbal therapy supplementations are the recent modifications done to improve oocyte retrieval and subsequent embryo transfer for POR cases. This review article presents the encouraging methods applied for POR cases to improve cycle outcome.

## 1. Introduction

The success of in vitro fertilization/intracytoplasmic sperm injection (IVF/ICSI) cycles are firstly dependent on the number of collected mature oocytes. Enough mature oocytes start the possibility of enough qualified embryos for transfer [1]. Low mature oocyte number due to decreased ovarian reserve (DOR) is one of the success-limiting factors for IVF/ICSI cycle outcomes [2]. Improving the cycle outcome is one of the struggle of infertility experts. 

It is known that advanced maternal age is a predictor of DOR. Surgical interventions, especially endometrioma extirpation from ovarian tissue, and chemotherapy, radiation therapy, and smoking are prominent factors decreasing ovarian follicular reserve. Genetic factors such as premature menopause or premature ovarian failure and follicle-stimulating hormone (FSH) receptor mutations are the other etiological factors of diminished ovarian oocyte pool [3,4].

In a group of IVF/ICSI cycles, despite appropriate ovarian stimulation, the number of oocytes collected is below the expected value. This condition is defined as poor ovarian response (POR) to stimulation. The incidence of poor responders in IVF/ICSI cycles approximately varies between 9% and 25% [5,6]. Poor response brings the risk of cycle cancellation with an estimated rate of 20% [7].

## 2. Definitions

Due to heterogeneous risk factors, there is not a distinct definition for POR. Researchers and committees have issued opinions for standardization. In 2011, The ESHRE consensus conference published the BOLOGNA criteria for definition of POR as the presence of two of the following criteria: 1) advanced maternal age (≥40 years) or any other risk factor for POR, 2) a previously characterized POR cycle (≤3 oocytes with a conventional stimulation protocol), 3) an abnormal ovarian reserve test (antral follicle count <5–7 follicles or AMH <0.5–1.1 ng/mL) [8].

Among the ovarian reserve tests, there are antral follicle count (AFC), FSH, anti-Müllerian hormone (AMH), inhibin B, and ovarian volume, but AMH, FSH, and AFC are the most sensitive ones [9]. In addition, two cycles with retrieval of three oocytes or less after maximal stimulation are enough to classify a patient as a poor responder, even in the absence of the other two criteria of BOLOGNA. Some researchers have criticized the BOLOGNA criteria for the heterogeneity of the patient population [8,10,11].  

Classification according to retrieved oocyte number brings four groups as follows: 1) Suboptimal response: retrieval of four to nine oocytes; 2) Normal responders: retrieval of 10–15 oocytes; 3) Hyperresponders: retrieval of more than 15 oocytes; 4) low responders: retrieval of fewer than 4 oocytes [12].

Recently the POSEIDON (Patient-Oriented Strategies Encompassing Individualized Oocyte Number) group reported a new approach for the definition and management of patients suffering from POR [13]. Their final aim was to determine the ideal stimulation for obtaining a euploid embryo for a successful transfer. This new approach classified the low responder women into four groups according to age, ovarian reserve, and stimulation response with the aim of determining the prognosis. 

**Group 1:** Patients younger than 35 with sufficient ovarian reserve parameters (AFC ≥5, AMH ≥1.2 ng/mL) and with an unexpected poor or suboptimal ovarian response;

Subgroup 1a:** <**4 oocytes retrieved.

Subgroup 1b: 4–9 oocytes retrieved.

**Group 2:** Patients older than 35 with sufficient ovarian reserve parameters (AFC >5, AMH >1.2 ng/mL) and with an unexpected poor or suboptimal ovarian response;

 Subgroup 2a: 4 oocytes retrieved.

 Subgroup 2b: 4–9 oocytes retrieved. 

**Group 3:** Patients younger than 35 with poor ovarian reserve parameters (AFC <5, AMH <1.2 ng/mL). 

**Group 4:** Patients older than 35 with poor ovarian reserve parameters (AFC <5, AMH <1.2 ng/mL). 

With this concept, low responders were defined as having poor prognosis. Age is the main predictor for IVF/ICSI cycle outcome because the older age brings DOR with decreased oocyte quality. Researchers observed lower pregnancy rates in older POR patients compared to that in younger POR patients [4].

## 3. Treatment modalities

Increasing gonadotropin doses in stimulation protocols is the first step used by all clinicians for poor responders. It was reported that there was no difference among 300–450 and 600 units of gonadotropins for IVF/ICSI cycle outcomes in poor responders [14]. It was accepted that long pituitary suppression with a GnRH agonist is detrimental for the oocyte pools of DOR cases. Due to this condition, microdose flare-up and short-flare protocols were developed for women suffering from POR [15]. 

Pituitary downregulation with GnRH antagonists is the second step to improve the cycle outcome in POR [16–18], but studies indicate that there is not a significant improvement in cycle outcomes with GnRH antagonists compared to agonist cycles [19–21].

The addition of growth hormones, transdermal testosterone, L-arginine, and pyridostigmine are experimental modifications that have been shown to not improve cycle outcomes in POR [22–25].

### 3.1. Stimulation modifications

Luteal estradiol (LE) priming is one of the other experimental modifications applied for POR to improve hypothalamic–pituitary–ovarian axis function [26]. Generally, LE priming is initiated on the 20th day of the previous cycle by daily administration of 4 mg of oral estradiol supplement or 0.1 mg of estradiol patch every other day, and is continued until day 2 of the following menstruation [27]. Supplementation of 4 mg of oral estradiol during the luteal phase combined with a short GnRH agonist protocol did not improve pregnancy rates compared to a long agonist protocol primed with oral contraceptive pills [28]. Metaanalysis showed that LE primed cycles had lower cancellation risk with improved clinical pregnancy rates compared to non-LE primed cycles despite no improvement on collected mature oocyte numbers and number of embryos per cycle [27].

Midfollicular recombinant luteinizing hormone (rLH) or urinary human chorionic gonadotrophin (HCG) supplementation is another experimental modification applied to improve retrieved oocytes in POR cases during antagonist cycles [29].

### 3.2. Double stimulation/Shanghai protocol

Researchers modify ovarian stimulation with a GnRH antagonist in different steps for POR. The first step is to combine gonadotropins with antiestrogenic agents such as clomiphene or letrozole. The second step is a GnRH agonist trigger combined with ibuprofen for final maturation before oocyte retrieval. For follicles with a diameter greater than 17 mm, oocytes are retrieved and embryo freezing is performed. The third step is luteal gonadotropin stimulation with an antiestrogenic agent with GnRH antagonist for follicles smaller than 13 mm in diameter. The fourth step is agonist trigger with ibuprofen again. The fifth step is endometrial preparation for frozen-thawed embryo transfer. This stimulation type gives the opportunity of more oocyte retrieval without improvement in live birth rate in POR [30,31].

### 3.3. Aromatase inhibitors

Letrozole is an aromatase inhibitor first applied for breast cancer for the decrement of estrogen levels. Decrement of estrogen levels results in increment of androgen levels. This microenvironment induces endogenous gonadotropin secretion and, according to this result, letrozole is being used for ovulation induction especially in POR [32]. Researchers reported improved cycle outcomes in gonadotropin dose decrement with letrozole combination compared to high-dose gonadotropin administration for POR [33].

## 4. Supplemental therapies

Dehydroepiandrosterone (DHEA) is a steroid prohormone originating from ovarian theca cells and the adrenal cortex [34]. DHEA is an androgenic supplement given to improve the number of oocytes collected in POR [35]. While some researchers reported improvement with DHEA supplementation on clinical pregnancy rates, live birth rate, endometrial thickness, and retrieved oocyte number [36], other researchers did not report improvement in cycle outcomes with DHEA supplementation [37].

The Kuntai capsule is one of the recent herbal therapy components of Chinese medicine applied for premature menopause. A Kuntai capsule consists of six traditional Chinese herbs, including Radix Rehmanniae Preparata, Rhizoma Coptidis, Radix Paeoniae Alba, Donkey Hide Gelatin, Radix Scutellariae, and Poria. In an experimental premature menopause model, researchers showed improvement in number of antral follicles with Kuntai capsule treatment [38]. Lian and Jing observed increment of retrieved oocyte numbers and high-quality embryos in POR cases after Kuntai capsule treatment [39]. 

## 5. Conclusion

Despite the multiple modifications of stimulation protocols and dietary intake presented here, POR remains a hard problem for infertility experts to solve.
